# Testicular Germ Cell Tumors in Children and Adolescents Treated With Bleomycin, Etoposide, and Cisplatin (BEP) Protocol: A Survival Analysis

**DOI:** 10.7759/cureus.48394

**Published:** 2023-11-06

**Authors:** Amaranto Suárez, Ma. Camila Prada-Avella, Eddie Pabón, Jorge L Buitrago, Jorge Hernández, Jhon Lopera, Mauricio Mesa, Alejandra Calderon, Luisa Barajas, Javier Muñoz, Martha Piña

**Affiliations:** 1 Pediatric Oncology, Instituto Nacional de Cancerología, Bogotá, COL; 2 Epidemiology, Instituto Nacional de Cancerología, Bogotá, COL

**Keywords:** survival, testicular, germ cell tumor, adolescents, children

## Abstract

Background: Germ cell tumors (GCTs) represent a diverse group of rare neoplasms that vary in location, histology, and clinical presentation. This study focuses on the clinical outcomes and survival rates of children and adolescents treated with the bleomycin, etoposide, and cisplatin (BEP) protocol.

Methods: This observational study evaluated children under 18 years diagnosed with testicular germ cell tumors and treated with the BEP protocol from January 2008 to December 2018. We employed descriptive analysis and used the Kaplan-Meier method to calculate event-free survival (EFS) and overall survival rates.

Results: The study included 32 patients with an average age of 9.8 years (SD ± 6.7). The primary reason for consultation was a testicular mass. The classification of patients was E-I for 14 patients (44%) and E-III and E-IV for nine patients (28%). Endodermal sinus tumors and mixed germ cell tumors were the most commonly identified histological types. With a median follow-up of 7.8 years (95% confidence interval {CI}: 5.9-9.6), the event-free survival was 63.7%. The overall survival at a median follow-up of 9.1 years (95% CI: 7.5-10.7) was 76.1%.

Conclusion: The BEP chemotherapy regimen offers promising results for treating testicular germ cell tumors in children and adolescents, characterized by its low toxicity and minimal late side effects. However, patients older than 11 years displayed more adverse histological indicators, advanced disease stages, and higher relapse and mortality rates.

## Introduction

Germ cell tumors (GCTs) are benign or malignant neoplasms originating from primordial germ cells. These tumors can be present in either gonadal or extragonadal locations. In children and adolescents under age 15, GCTs constitute 2%-3% of all diagnosed cancers. However, in the age range of 15-19 years, the incidence rises to as high as 15%, positioning them as the third most common cancer after thyroid cancer and Hodgkin’s lymphoma [1]. The incidence rate is 0.5-2 cases per million children under age 15 [2].

Histologically, GCTs vary and include mature and immature teratomas, endodermal sinus tumors (also known as yolk sac tumors), embryonal carcinoma, choriocarcinoma, seminoma, dysgerminoma, and germinoma [3,4]. Regardless of the histological type or patient’s age, testicular GCTs typically present clinically as a nonpainful mass that might appear as scrotal inflammation. If the tumor extends to the spermatic cord, it can be mistaken for epididymitis.

Over the past four decades, malignant GCTs have become treatable primarily due to their high sensitivity to various antineoplastic drugs. The introduction of cisplatin into multi-agent treatment protocols for testicular tumors, including cisplatin, vinblastine, and bleomycin (PVB), has led to complete and lasting remissions in 70%-90% of newly diagnosed patients [5]. In the mid-1980s, a clinical trial involving adult male GCT patients with disseminated disease showed that replacing vinblastine with etoposide in the PVB regimen resulted in fewer side effects and increased efficacy [6]. As a result, the bleomycin, etoposide, and cisplatin (BEP) protocol became the standard treatment for GCTs, especially testicular GCTs.

The original BEP protocol underwent minor modifications in pediatric treatments to reduce toxicity and late side effects. Notably, the frequency of bleomycin administration was reduced from weekly to once every three weeks to safeguard children’s immature lungs from its toxic effects. This adaptation is termed the pediatric BEP or BEP protocol [7]. We conducted this study to assess the demographics, clinical characteristics, and survival rates of a group of children and adolescents with testicular GCTs treated using the BEP protocol at the Instituto Nacional de Cancerología (INC).

## Materials and methods

We conducted a retrospective cohort observational study with survival analysis. The study included all patients under 18 years diagnosed with testicular GCTs, such as seminoma, endodermal sinus tumor, choriocarcinoma, embryonal carcinoma, or teratoma, confirmed by either histopathology or elevated serum levels of tumor markers (alpha-fetoprotein {AFP} and/or beta-subunit of chorionic gonadotrophin hormone). All patients received the BEP chemotherapy regimen at the pediatric oncology unit of the INC, Bogotá, Colombia, between January 1, 2008, and December 31, 2018. All underwent orchiectomy. For patients who had orchiectomy at different institutions, we conducted a pathology review of their specimens. We excluded patients treated with chemotherapy or radiotherapy at other institutions and those with extragonadal primary tumors. All relevant clinical, surgical, radiological, and laboratory data were extracted from medical records. The ethics and research committee of the INC of Colombia approved this study.

At diagnosis, every patient underwent abdominopelvic and chest computed tomography, blood count, and tests for transaminases, bilirubin, creatinine, serum urea nitrogen, and serum electrolytes (Na, Cl, K, Ca, P, and Mg). We also assessed tumor markers, including lactate dehydrogenase, alpha-fetoprotein (AFP), and beta-human chorionic gonadotropin (β-hCG). Staging followed the Children’s Oncology Group (COG) staging system, as outlined in Table 1.

**Table 1:** Testicular GCT staging from the COG in children and adolescents COG, Children’s Oncology Group; CT, computed tomography; GCT, germ cell tumor

Stage	Extent of Disease
I	Tumor limited to the testis (testes) with negative microscopic margins, completely resected by high inguinal orchiectomy. No clinical, radiographic, or histological evidence of disease beyond the testes. Normal tumor markers after appropriate postsurgical half-life decline time (patients with normal or unknown markers at diagnosis should have a negative ipsilateral retroperitoneal node dissection to confirm stage I disease if imaging demonstrates lymph nodes of >2 cm)
II	Transscrotal biopsy; transscrotal orchiectomy with macroscopic tumor spillage. A microscopic disease of the scrotum or in the high spermatic cord (<5 cm from the proximal border). Retroperitoneal lymph node involvement (≤2 cm). Positive tumor markers after the adequate time of half-life decline
III	Retroperitoneal lymph node involvement but no visceral or extra-abdominal involvement. Lymph nodes of ≥2 cm or lymph nodes of >1 cm but <2 cm by multiplanar imaging CT that fail to resolve on reimaging at 4-6 weeks
IV	Distant metastases, including the liver, lung, bone, and brain

All participants received the BEP chemotherapy protocol: cisplatin (100 mg/m^2^, day 1), etoposide (100 mg/m^2^, days 1-5), and bleomycin (15 IU/m^2^, day 1). Treatment cycles were repeated every 21-28 days based on hematologic and renal tolerance. Before starting each chemotherapy cycle, we conducted tests including hemogram, transaminases, bilirubin, serum urea nitrogen, creatinine, serum electrolytes, AFP, β-hCG, glomerular filtration scintigraphy, and abdominopelvic ultrasound.

Treatment varied according to stage: Stage I patients with teratoma or endodermal sinus tumors were observed, stage I patients with malignant components received two cycles, stage III patients had four cycles, and stage IV patients underwent six cycles. After treatment, follow-ups included physical examinations and tests for blood count, serum electrolytes, alpha-fetoprotein, and β-hCG and abdominopelvic ultrasound every two months for the initial six months. This frequency then shifted to every four months up to two years posttreatment and annually after that.

We used absolute and relative frequency distributions for categorical variables for data analysis. The Kaplan-Meier method determined the time to relapse or death. Event-free survival (EFS) and overall survival were defined as the time from diagnosis to the first relapse, progression, or death and the time from diagnosis to any cause of death, respectively. Patients without events were censored. Statistical analyses were two-tailed with a 0.05 type I error level, using the Statistical Package for Social Sciences (SPSS) version 20.0 (IBM SPSS Statistics, Armonk, NY).

## Results

Between January 1, 2008, and December 31, 2018, we diagnosed 70 patients with gonadal GCTs. Of these, 32 were testicular tumors, with an average age of 9.8 years (SD ± 6.7). Fifteen patients (47%) were under 11 years old, while 17 (53%) were 11 or older. The most common symptom was a painless testicular mass, observed in 28 (88%) of the 32 patients. Abdominal pain was the second most common symptom, occurring in 10 patients (30.1%).

In terms of disease staging, 14 patients (44%) presented with stage E-I disease, nine (28%) with stage III, and nine (28%) with stage E-IV. No patients were classified as E-II. The metastatic disease appeared as single retroperitoneal lymph node involvement in nine patients (28%). Another three patients (9.4%) had isolated pulmonary metastases, while six (19%) displayed combined metastases in retroperitoneal lymph nodes and bone, liver, or lung involvement.

Of the tumors, three cases (9.4%) were nonmalignant (comprising two mature teratomas and one mixed with both mature and immature teratoma components). The remaining 29 cases were malignant tumors of varying histologies. Table 2 provides a detailed account of clinical and histological characteristics, staging, and outcomes. We performed inguinal orchiectomies on 30 patients (94%). Two patients had scrotal orchiectomies at outside institutions; both were staged as III due to retroperitoneal lymph node involvement.

**Table 2:** Clinical features of testicular GCT EST, endodermal sinus tumor; GCT, germ cell tumor; COG, Children’s Oncology Group

Features	All, n = 32	<11 Years	≥11 Years
Age in years (± SD)	9.8 (± 6.7)	3.0 (± 2.6)	15.7 (± 1.64)
Clinical manifestation, n (%)			
Testicular mass, n (%)	28 (88)	15	13
Abdominal pain, n (%)	10 (31)	2	8
Abdominal mass, n (%)	2 (6)	0	2
Lumbar pain, n (%)	2 (6)	0	2
Dyspnea, n (%)	1 (3)	0	1
Surgery, n (%)	32 (100)	15 (47)	17 (53)
Inguinal orchiectomy, n (%)	30 (94.0)	14 (93)	16 (94)
Scrotal orchiectomy, n (%)	2 (6.0)	1 (7)	1 (6)
Retroperitoneal ganglion dissection, n (%)	1 (3.1)		
Histological diagnosis, n (%)	32 (100)	15 (47)	17 (53)
Mature teratoma, n (%)	2 (6)	1 (7)	1 (6)
Immature teratoma, n (%)	0	0	0
Nonmalignant mixed teratoma, n (%)	1 (3)	0	1 (6)
EST, n (%)	13 (41)	13 (86)	0
Embryonal carcinoma, n (%)	1 (3)	0	1 (6)
Choriocarcinoma, n (%)	1 (3)	0	1 (6)
Mixed malignant GCT, n (%)	13 (41)	1 (7)	12 (70)
Seminoma, n (%)	1 (3)	0	1 (6)
COG staging at diagnosis, n (%)	32 (100)	15 (47)	17 (53)
Stage I, n (%)	14 (44)	11 (73)	3 (18)
Stage II, n (%)	0	0	0
Stage III, n (%)	9 (28)	3 (20)	6 (35)
Stage IV, n (%)	9 (28)	1 (7)	8 (47)
Events, n (%)	10 (100)	2 (20)	8 (80)
Relapses	4 (12.5)	0	4 (50)
Progression	3 (9.4)	2 (100)	1 (12)
Death	3 (9.4)	0	3 (38)

Table 2 also presents the clinical outcomes: of the 32 patients, 10 (31%) experienced events. This includes four relapses, three cases of progressive disease, and six deaths. All the relapsed patients and all deaths were in the age group of 11 years and older. Patients generally tolerated the BEP protocol well. We observed no deaths attributable to the treatment or second malignancies. Furthermore, no patient showed pulmonary involvement from bleomycin or renal toxicity from cisplatin up to the date of observation closure.

**Figure 1:**
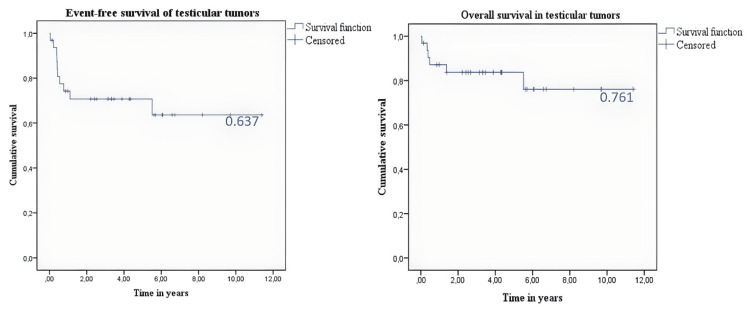
(Left panel) Event-free survival. (Right panel) Overall survival

**Figure 2:**
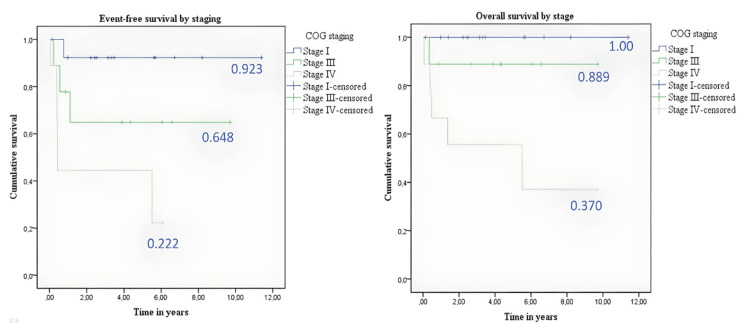
(Left panel) Event-free survival by stage. (Right panel) Overall survival by stage COG: Children’s Oncology Group

The median follow-up was 7.8 years (95% confidence interval {CI}: 5.9-9.6) for EFS, which stood at 63.7%. The overall survival rate was 76.1%, with a median follow-up of 9.1 years (95% CI: 7.5-10.7). For a more detailed breakdown of EFS and overall survival by disease stage, refer to Table 3, Figure 1, and Figure 2.

**Table 3:** Event-free and overall survival of testicular GCTs GCTs, germ cell tumors; IC, information coefficient

	Survival	Mean Time
Event-free survival (IC 95%)	63.7	7.8 (5.9-9.6)
Stage I	92.3	
Stage III	64.8	
Stage IV	22.2	
Overall survival (IC 95%)	76.1	9.1 (7.5-10.7)
Stage I	100	
Stage III	88.9	
Stage IV	37	

## Discussion

Gonadal and extragonadal GCTs are rare neoplasms in the pediatric age group. They have an incidence rate of 2.4 cases per million children and account for approximately 2%-3% of cancers diagnosed in children under 15 years. Interestingly, their prevalence increases with age, comprising up to 15% of all cancers diagnosed between ages 15 and 19. The distribution of GCTs in children follows a bimodal pattern, with initial peaks from infancy to four years and another peak at puberty onset, each with distinct clinical and molecular features [1,8,9].

We observed a similar bimodal distribution for testicular GCTs: a peak from birth to three years, predominantly featuring teratomas and yolk sac (endodermal sinus) tumors, and another peak starting at age 12, peaking at 15 years. This latter group predominantly displayed mixed GCTs with a higher proportion of embryonal carcinomas and choriocarcinomas. Even though our study had a small sample size, the age groups align with other reports [2,7,10,11]. For patients younger than 11 years, the average age was three years, with most having endodermal sinus tumors. In contrast, for those older than 11 years, the average age was 15.7 years, with mixed malignant germ cell tumors as the predominant histology. We also noticed that adolescents had a higher proportion of advanced disease (56% for stages E-III and E-IV combined) than the younger group (44%).

Supporting our findings, Aldrink et al. [11] reported on testicular GCTs, and Calaminus et al. [12] discussed the Maligne Keimzelltumoren (MAKEI) 96 protocol’s clinical outcomes for 1465 patients under 18 with ovarian and testicular GCTs. They emphasized that patients aged 11 or older, especially those with mixed or embryonal carcinoma histology and stage III or IV or those with positive lymphovascular invasion, faced higher relapse rates and mortality [13,14]. In our series, we found that five of the six deceased patients were 15 years or older, with four having mixed GCT histology and one having choriocarcinoma, and five were classified as stage IV and one as stage III (Table 4).

**Table 4:** Deaths in children and adolescents with testicular GCTs GCTs, germ cell tumors; RLNI, retroperitoneal lymph node involvement; EST, endodermal sinus tumor

Age (Years)	Surgery	Stage	Metastases	Histology	Chemotherapy	Cycles (Number)
16	Inguinal	IV	RLNI + lung	Mixed	Adjuvant	5
17	Inguinal	IV	Lung	Mixed	Neoadjuvant	2
3	Inguinal	IV	Lung	EST	Adjuvant	6
17	Scrotal	III	RLNI	Mixed	Adjuvant	5
16	Inguinal	IV	Lung	Mixed	Adjuvant	1
15	Inguinal	IV	RLNI + lung	Choriocarcinoma	Adjuvant	1

Historically, chemotherapy treatments for malignant GCTs have often mirrored those for testicular cancer. Treatment outcomes began to improve in the late 1970s following cisplatin’s introduction, leading to disease-free survival rates ranging from 68% to 92% [5,15,16]. The COG and the pediatric germ cell tumor intergroup have shown impressive six-year EFS and overall survival rates for testicular tumors [17,18]. However, our cohort’s results were somewhat less favorable than those reported by developed countries or regional counterparts [19,20]. As evident in Table 4, most deaths in our series occurred in patients older than 11 years with stage IV disease, and apart from one three-year-old patient, tumor histology was primarily mixed germ cell, known for its poorer prognosis. We believe that late diagnoses due to delayed referrals and interventions at nonspecialized centers and socioeconomic constraints could adversely affect outcomes.

Our study has several important limitations. Chief among them is its retrospective design, which inherently brings forth potential biases, including selection bias and missing or incomplete data. The relatively small sample size might limit the generalizability of our findings to a broader population. Additionally, our dataset’s lack of genetic and molecular analyses hinders a deeper understanding of the tumor profiles and their potential influence on outcomes. While we have highlighted possible systemic and socioeconomic factors that could affect diagnostic and treatment timelines, a comprehensive evaluation of these factors was beyond the scope of our study. Thus, caution is advised when extrapolating our findings to different populations or healthcare settings. However, our results highlight the potential to incorporate genetic and molecular studies in future cohorts, refine risk group classifications, and enhance supportive care to prevent unnecessary deaths.

## Conclusions

Testicular GCTs in children and adolescents are treatable diseases, with chemotherapy regimens that exhibit low toxicity and minimal long-term side effects. Patients older than 11 years tend to have adverse histological features, advanced disease stages, and a higher incidence of relapses and mortality. Some younger patients with stage I GCTs and endodermal sinus tumors might be effectively managed with surgery and observation. Given GCTs’ low incidence, specialized pediatric cancer centers should treat all patients. Integrating GCT patients into standardized treatments with established multi-institutional outcomes is essential.
